# Prevalence and Correlates of Dilated and Non-Dilated Left Ventricular Cardiomyopathy in Transfusion-Dependent Thalassemia: Data from a National, Multicenter, Observational Registry

**DOI:** 10.3390/jcdd12030103

**Published:** 2025-03-16

**Authors:** Antonella Meloni, Laura Pistoia, Anna Spasiano, Francesco Sorrentino, Giuseppe Messina, Michele Santodirocco, Zelia Borsellino, Valerio Cecinati, Vincenzo Positano, Gennaro Restaino, Nicolò Schicchi, Emanuele Grassedonio, Antonino Vallone, Michele Emdin, Alberto Clemente, Andrea Barison

**Affiliations:** 1 Bioengineering Unit, Fondazione G. Monasterio CNR-Regione Toscana, 56124 Pisa, Italy; antonella.meloni@ftgm.it (A.M.); positano@ftgm.it (V.P.); 2 Unità Operativa Semplice Dipartimentale Ricerca Clinica, Fondazione G. Monasterio CNR-Regione Toscana, 56124 Pisa, Italy; laura.pistoia@ftgm.it; 3 Unità Operativa Semplice Dipartimentale Malattie Rare del Globulo Rosso, Azienda Ospedaliera di Rilievo Nazionale “A. Cardarelli”, 80131 Napoli, Italy; spasiano.anna@tiscali.it; 4 Unità Operativa Semplice Dipartimentale Day Hospital Talassemici, Ospedale “Sant’Eugenio”, 00143 Roma, Italy; sorrentino.francesco@aslrmc.it; 5 Centro Microcitemie, Grande Ospedale Metropolitano “Bianchi-Melacrino-Morelli”, 89100 Reggio Calabria, Italy; gspmessina@virgilio.it; 6 Centro Microcitemia—Day Hospital Thalassemia Poliambulatorio “Giovanni Paolo II”, Istituto di Ricovero e Cura a Carattere Scientifico Ospedale Casa Sollievo della Sofferenza, 71013 San Giovanni Rotondo, Italy; m.santodirocco@operapadrepio.it; 7 Unità Operativa Complessa Ematologia con Talassemia, Azienda di Rilievo Nazionale ad Alta Specializzazione Civico “Benfratelli-Di Cristina”, 90134 Palermo, Italy; zelia.borsellino@arnascivico.it; 8 Struttura Semplice di Microcitemia, Ospedale “SS. Annunziata”, 74123 Taranto, Italy; valerio.cecinati@als.taranto.it; 9 Radiology Department, Responsible Research Hospital, 86100 Campobasso, Italy; gennaro.restaino@responsible.hospital; 10 Dipartimento di Radiologia, Azienda Ospedaliero-Universitaria Ospedali Riuniti “Umberto I-Lancisi-Salesi”, 60020 Ancona, Italy; schicchi.n@alice.it; 11 Sezione di Scienze Radiologiche—Dipartimento di Biopatologia e Biotecnologie Mediche, Policlinico “Paolo Giaccone”, 90127 Palermo, Italy; egrassedonio@gmail.com; 12 Reparto di Radiologia, Azienda Ospedaliera “Garibaldi” Presidio Ospedaliero Nesima, 95126 Catania, Italy; ninovallone@hotmail.com; 13 Cardiology and Cardiovascular Medicine, Fondazione G. Monasterio CNR-Regione Toscana, 56124 Pisa, Italy; emdin@ftgm.it; 14 Institute of Life Sciences, Scuola Superiore Sant’Anna, 56124 Pisa, Italy; 15 Department of Radiology, Fondazione G. Monasterio CNR-Regione Toscana, 56124 Pisa, Italy; clemente@ftgm.it

**Keywords:** transfusion-dependent β-thalassemia, cardiovascular magnetic resonance, dilated cardiomyopathy, non-dilated left ventricular cardiomyopathy, prognosis

## Abstract

We investigated the prevalence, clinical characteristics, and prognostic role of dilated cardiomyopathy (DCM) and non-dilated left ventricular cardiomyopathy (NDLVC) in patients with transfusion-dependent β-thalassemia (β-TDT). We retrospectively included 415 β-TDT patients who underwent cardiovascular magnetic resonance to quantify myocardial iron overload (MIO) and biventricular function parameters and to detect replacement myocardial fibrosis. Demographic and laboratory parameters were comparable among patients with no overt cardiomyopathy (NOCM; *n* = 294), DCM (*n* = 12), and NDLVC (*n* = 109), while cardiac size and systolic function were significantly different. Compared to NOCM patients, DCM and NDLVC patients had a higher prevalence of MIO and replacement myocardial fibrosis. During a mean follow-up of 57.03 ± 18.01 months, cardiac complications occurred in 32 (7.7%) patients: 15 heart failures, 15 supraventricular arrhythmias, and 2 pulmonary hypertensions. Compared to the NOCM group, both the NDLVC and the DCM groups were associated with a significantly increased risk of cardiac complications (hazard ratio = 4.26 and 8.81, respectively). In the multivariate analysis, the independent predictive factors were age, MIO, and the presence of DCM and NDLVC versus the NOCM phenotype. In β-TDT, the detection of NDLVC and DCM phenotypes may hold value in predicting cardiac outcomes.

## 1. Introduction

Thalassemia syndromes represent the most common autosomal recessively inherited blood disorder worldwide. Thalassemia syndromes are most prevalent in the Mediterranean, Middle East, Indian subcontinent, and Southeast Asia, but migration patterns have altered their global distribution, making them increasingly common in regions where they were historically rare, such as Western Europe and North America [1,2]. Beta-thalassemia results from mutations in the β-globin gene on chromosome 11, leading to insufficient or absent production of the β-globin chain. This deficiency disrupts hemoglobin synthesis, causing ineffective erythropoiesis, hemolysis, and anemia [3,4,5]. The most severe clinical form of β-thalassemia is transfusion-dependent β-thalassemia (β-TDT), characterized by a lifelong dependency on regular blood transfusions since early childhood to manage symptoms and sustain life [6,7,8]. Chronic transfusions inevitably lead to iron accumulation, as the human body lacks a natural excretory pathway for excess iron [9,10,11]. Excess iron deposits in critical organs, including the heart, liver, and endocrine glands, leading to dysfunction or failure [12,13,14]. Cardiac complications are the primary cause of death among TDT patients [15,16,17]. A significant improvement in outcomes and survival has been achieved thanks to the introduction of iron chelation therapy and the cardiovascular magnetic resonance (CMR) T2* technique [16,18,19]. T2* CMR has become a key noninvasive tool for assessing myocardial iron levels and personalizing and assessing the efficacy of chelation therapy [20,21,22,23,24].

In addition to myocardial iron overload (MIO), several other factors, including chronic anemia, myocarditis, endocrine abnormalities, and genetic factors, can contribute to the development of cardiac complications [17,25,26,27,28,29,30]. Despite ongoing transfusion therapy, TDT patients remain characterized by chronic anemia, which can cause a high-output state, where the heart compensates for decreased oxygen-carrying capacity by sustaining an elevated cardiac output. The prolonged volume overload leads to cardiac remodeling, with dilation of the chambers and thickening of the ventricular walls, significantly raising the risk of heart dysfunction or even failure [31,32,33]. So, the accurate and reproducible assessment of biventricular volumes and function is crucial in the cardiac management of TDT patients, and CMR represents the gold standard modality for this purpose [34,35,36]. To accurately detect cardiac impairment and avoid misdiagnosis (overdiagnosis of dilatation and underdiagnosis of dysfunction), specific CMR reference ranges for TDT patients should be used rather than the standard limits used for the general population [37,38,39].

Another ability of CMR, crucial for the diagnosis and prognostic stratification of left-sided cardiomyopathies, is the detection of myocardial fibrosis or scar. Replacement myocardial fibrosis has emerged as a strong independent predictor of major cardiovascular events in both thalassemic [40,41] and non-thalassemic populations [27,42,43,44,45].

In TDT, there are a lack of data about the effective prevalence, the clinical correlates, and the prognostic implications of the dilated cardiomyopathy (DCM) phenotype, characterized by the dilatation of the LV accompanied by systolic dysfunction in the absence of significant coronary artery disease (CAD) [46]. Moreover, the European Society of Cardiology (ESC) has recently proposed a new clinical definition of cardiomyopathy called non-dilated left ventricular cardiomyopathy (NDLVC), characterized by the absence of LV dilatation and the presence of LV non-ischemic fibro-fatty replacement regardless of the presence of wall motion abnormalities, and/or isolated global LV systolic dysfunction [47].

The aim of this multicenter study was to investigate the prevalence, clinical characteristics, and prognosis in terms of cardiovascular events of DCM and NDLVC in patients with β-TDT.

## 2. Materials and Methods

### 2.1. Study Population

We retrospectively selected 425 β-TDT patients who performed their first CMR scan for the simultaneous assessment of cardiac iron overload and function within the Myocardial Iron Overload in Thalassemia (MIOT) project, which started in 2006 and lasted more than 10 years [16]. MIOT was an Italian network consisting of 70 thalassemia centers enrolling patients and 10 magnetic resonance imaging (MRI) facilities performing CMR examinations according to validated and standardized protocols [48]. All centers were linked by a web-based database, where the clinical-anamnestic history of the patients, from birth to the date of the first CMR, was recorded. The clinical, instrumental, and laboratory data were updated at every CMR scan, performed by protocol every 18 ± 3 months.

All patients included in this study fulfilled the general inclusion criteria of the MIOT project: (1) male and female patients of any age with thalassemia syndromes or structural hemoglobin variants requiring MRI for cardiac and liver iron burden assessment; (2) provision of written informed consent; (3) written authorization for the use and disclosure of protected health information; and (4) absence of absolute contraindications to MRI. Missing clinical data, previous history of ischemic heart disease, and presence of a cardiac complication (heart failure, arrythmias, and pulmonary hypertension) developed before the enrolment in the MIOT project and still active at the baseline CMR represented the exclusion criteria for this retrospective analysis evaluation.

All patients received regular blood transfusions since early childhood, to keep their pre-transfusion hemoglobin levels above 9–10 g/dL, and iron chelation therapy prescribed based on the current clinical practice according to clinical, laboratory, and instrumental data.

The study complied with the Declaration of Helsinki and was approved by the ethical committees of all the MRI sites involved in the study. All patients gave written informed consent.

### 2.2. CMR Protocol

CMR scans were conducted within one week prior to the scheduled transfusion sessions.

All patients underwent CMR using conventional clinical 1.5 T scanners from three main vendors (GE Healthcare, Milwaukee, WI, USA; Philips, Best, Netherlands; Siemens, Erlangen, Germany). All images were acquired during breath holding in end-expiration and using electrocardiographic gating.

For the assessment of cardiac anatomy and ventricular function, steady-state free precession (SSFP) cine sequences were acquired in two-, three- and four-chamber planes and short axis slices (slice thickness 8 mm, no gap) covering the ventricle over its entire extension [39]. Thirty cardiac phases were acquired per heartbeat. Manual post-processing was performed by expert operators (>10 years of experience in CMR) using a commercially available software system (MASS, Medis, Leiden, The Netherlands, or cmr42, Circle Cardiovascular Imaging Inc., Calgary, AB, Canada). Intra- and inter-observer reproducibility of biventricular size and function measurements in TM patients was shown to be high [49]. Left ventricular (LV) and right ventricular (RV) volumes were quantified from the series of short-axis cine images, with the analysis based on the manual definition of the endocardial and epicardial borders at both end-diastolic and end-systolic phases for each slice. The papillary muscles were also outlined and included as part of the myocardial mass rather than the blood pool. End-diastolic volume (EDV) and end-systolic volume (ESV) were calculated using Simpson’s rule, avoiding the need for geometric assumptions regarding ventricular shape [50]. LV mass was determined by multiplying the myocardial volume by its specific density of 1.05 g/cm^3^. Biventricular volumes and LV mass were indexed to the body surface area (BSA), derived using the variation of the Dubois and Dubois formula [51]. The stroke volume index (SVI) was calculated as the difference between EDV index (EDVI) and ESV index (ESVI). The ejection fraction (EF) was given by the ratio between the SVI and the EDVI.

Left and right atrial areas were measured from the four-chamber view projection in the ventricular end-systolic phase and were normalized by BSA.

Presence of myocardial fatty replacement was defined by the presence of “India Ink” (banding) artifact within the LV myocardium in cine SSFP images, confirmed by two orthogonal planes [52].

For the assessment of MIO, three parallel short-axis views (basal, medium, and apical) of the LV were acquired at ten echo times by a T2* gradient–echo multiecho sequence [53]. Image analysis was performed using a custom-written, previously validated software (HIPPO MIOT^®^ Version 2.0, Consiglio Nazionale delle Ricerche and Fondazione Toscana Gabriele Monasterio, Pisa, Italy) [54]. The myocardial T2* distribution was mapped into a 16-segment LV model, according to the AHA/ACC model [55]. An appropriate correction map was applied to correct the susceptibility artifacts [54]. The global heart T2* value was obtained by averaging all segmental T2* values. The T2* segmental approach demonstrated good inter-site, intra-study, intra-observer, and inter-observer variability [48] and was validated against autoptic hearts [56].

To detect replacement/focal myocardial fibrosis, short-axis and long-axis late gadolinium enhancement (LGE) images were acquired by a T1-weighted gradient-echo inversion-recovery pulse sequence, 8–18 min after the intravenous administration of Gadobutrol (Gadovist^®^; Bayer Schering Pharma; Berlin, Germany) at the standard dose of 0.2 mmol/kg of body weight [40]. Images were evaluated qualitatively for the presence, pattern (ischemic or non-ischemic), and regional distribution of LGE areas [40,57]. LGE was considered present if visualized in two different views. The LGE pattern was considered ischemic if it was subendocardial or transmural in the territory of a coronary artery; otherwise, it was classified as a nonischemic pattern. The extent of LGE areas was quantified as a percentage of the LV mass using a validated software [58].

### 2.3. Laboratory Investigation

All biochemical tests were conducted using commercially available kits at the laboratories of the thalassemia centers where the patients were treated.

The average value of hemoglobin and ferritin levels over the last 12 months preceding the CMR was considered.

### 2.4. Diagnostic Criteria

LV dilation was diagnosed in the presence of LV EDVI higher than previously derived cut-offs, specific to TM patients and stratified by age and gender [39]. LV dysfunction was diagnosed in the presence of LV EF lower than previously derived cut-offs, specific to TM patients and stratified by age and gender [39].

According to the 2023 [47] guidelines developed by the ESC, three groups of patients were defined as follows.

- DCM: presence of LV dilatation and systolic dysfunction, unexplained by congenital, valvular, hypertensive, or coronary heart diseases. Non-ischemic LGE may be absent or present. Right ventricular dilatation and dysfunction may be present but were not included in the diagnosis.
- NDLVC: presence of non-ischemic LGE, fatty replacement, or global LV systolic dysfunction in the absence of LV dilatation.
- NOCM (no overt cardiomyopathy): absence of LV systolic dysfunction, LGE, and fatty replacement areas.

Diabetes mellitus was defined as fasting plasma glucose ≥ 126 mg/dL or 2-h plasma glucose ≥ 200 mg/dL during an oral glucose tolerance test or random plasma glucose ≥ 200 mg/dL with classic symptoms of hyperglycemia [59].

A T2* measurement of 20 ms was the “conservative” normal value for the segmental and global heart T2* values [54,60]. A global heart T2* < 20 ms indicated significant MIO.

### 2.5. Clinical Follow-Up and Outcomes

The end of the follow-up coincided with the date of the last available complete clinical and imaging evaluation. For patients who did not perform a follow-up CMR evaluation, a case report form detailing patient outcomes between the baseline CMR exam and the end of the project was completed by the caring thalassemia center.

The following events were considered: heart failure (HF), ventricular or supraventricular arrhythmias, and pulmonary hypertension (PH). HF was diagnosed based on symptoms (i.e., fatigue, breathlessness, ankle swelling), signs, and instrumental findings, in accordance with current guidelines [61]. Arrhythmias were diagnosed if recorded by ECG or 24-h Holter monitoring and necessitating specific treatments and were classified according to the AHA/ACC guidelines [62]. PH was diagnosed if the trans-tricuspidal velocity jet by echocardiography was greater than 3.2 m/s [63]. If a patient developed more than one complication during the follow-up, only the first one was considered.

### 2.6. Statistical Analysis

Statistical analyses were performed using SPSS version 27.0 statistical package (IBM Corp, Armonk, NY, USA).

Continuous variables were described as mean ± standard deviation (SD), and categorical variables were expressed as frequencies and percentages.

The normality of the distribution of the parameters was assessed by using the Kolmogorov-Smirnov test.

For continuous values with normal distribution, comparisons between groups were made by independent-sample *t*-test (for 2 groups) or one-way ANOVA (for > 2 groups). The Mann–Whitney U test or Kruskal–Wallis test were applied for continuous values with non-normal distribution. The χ^2^ testing was used to compare categorical variables. The Bonferroni post hoc test was used for multiple comparisons between pairs of groups.

The Cox proportional-hazard model was used to test the association between the considered prognostic variables (demographics, cardiovascular risk factors, CMR parameters) and the outcome. All variables showing a significant association in the univariate model were placed in the multivariate model and were ruled out only if they did not significantly improve the adjustment of the model. The results were presented as hazard ratio (HR) with 95% confidence intervals (CI). Kaplan–Meier curves were generated by relating the development of an outcome over time to CMR findings. The log-rank test was used to compare different strata in Kaplan–Meier analyses.

Two-sided *p*-values were calculated in all tests, and statistical significance was defined as *p* < 0.05.

## 3. Results

### 3.1. Patient Data

TDT patients had a mean age of 29.5 ± 8.9 years, and 228 (53.6%) were females. Mean LVEF was 62.4 ± 6.1% while mean LV EDVI was 85.96 ± 18.59 mL/m^2^. No patients presented isolated fatty replacement areas within the LV myocardium.

Twelve (2.8%) patients had DCM and 109 (25.6%) patients had NDLVC. Among the NDLVC patients, 43 (39.5%) presented isolated LV systolic dysfunction, 60 (55.0%) isolated LGE, and 6 (5.5%) both LV dysfunction and LGE.

The NOCM group was constituted of 304 (71.5%) patients. Ten patients in this group had isolated LV dilatation (LV dilatation with normal LV EF) and were excluded from the analysis to avoid any bias. So, the final NOCM group was composed of 294 patients.

**Figure 1:**
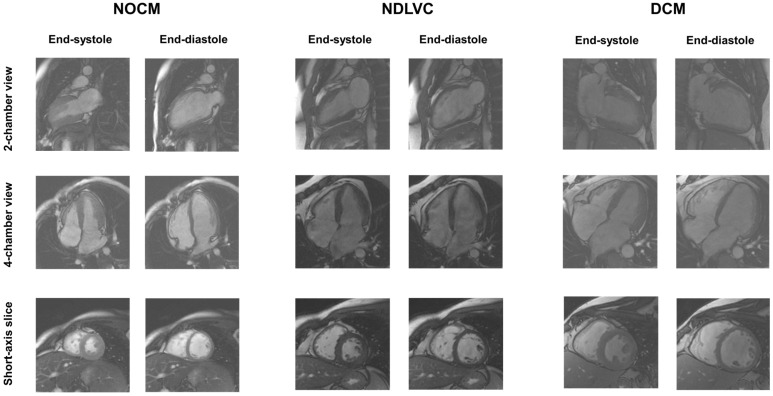
Example of cine images (2-chamber view, 4-chamber view, and short-axis slice) in end-systolic and end-diastolic phases for a TDT patient with normal LV size and function (**left**), a TDT patient with NDLVC (**center**), and a TDT patient with DCM (**right**). NOCM = no overt cardiomyopathy, NDLVC = non-dilated left ventricular cardiomyopathy, DCM = dilated cardiomyopathy.

Representative cine images for one patient in each group are shown in Figure 1.

### 3.2. Demographic and Clinical Characteristics of TDT Patients Stratified Based on Cardiac Phenotype

Table 1 shows the comparison of demographic, hematochemical, and clinical characteristics among the three groups of TDT patients identified according to the cardiac phenotype (NOCM, NDLVC, and DCM). No significant differences were found in terms of age, sex, starting age of regular transfusions or chelation, mean pre-transfusion hemoglobin and ferritin levels, prevalence of diabetes, active or past hepatitis C virus (HCV) infection, and overweight.

Splenectomy was significantly more frequent in the DCM group compared to both the NOCM and the NDLVC groups (*p* = 0.003 and *p* = 0.024, respectively). The age of splenectomy was comparable among the three groups.

### 3.3. Myocardial Iron Overload and Cardiac Phenotype

Cardiac iron levels were significantly different among the three groups. Specifically, compared to the NOCM group, both DCM and NDLVC patients showed significantly lower global heart T2* values (*p* = 0.024 and *p* = 0.006, respectively) and a significantly higher number of segments with T2* < 20 ms (*p* = 0.027 and *p* = 0.012, respectively). The prevalence of significant MIO was increased in the DCM group compared to the NOCM group (*p* = 0.006).

### 3.4. CMR Findings and Cardiac Phenotype

Table 1 shows the comparison of CMR parameters among the three groups of TDT patients identified according to the cardiac phenotype.

LV EDVI and ESVI were significantly increased in the DCM group compared to both NOCM and NDLVC groups (*p* < 0.0001 for all comparisons) and in the NDLVC group compared to the NOCM group (LV EDVI: *p* = 0.012 and LVESVI: *p* < 0.0001). DCM patients exhibited significantly higher LV SVI and mass index than the NOCM group (*p* < 0.0001 for both comparisons) and the NDLVC group (LVSVI: *p* < 0.0001 and LV mass index: *p* < 0.0001). The LV EF was significantly lower in the DCM group compared to both the NOCM and the NDLVC groups (*p* < 0.0001 and *p* = 0.006, respectively) and in the NDLVC group compared to the NOCM group (*p* < 0.0001).

RV EDVI, ESVI, and SVI were significantly increased in the DCM group compared to both NOCM and NDLVC groups (*p* < 0.0001 for all comparisons). The RV ESVI was also significantly different between the NDLVC and NOCM groups (*p* < 0.0001). The RV EF was significantly lower in the DCM group compared to both the NOCM and the NDLVC groups (*p* < 0.0001 and *p* = 0.018, respectively) and in the NDLVC group compared to the NOCM group (*p* < 0.0001).

Both left and atrial areas were significantly higher in the DCM group compared to the NOCM group (*p* < 0.0001 for both comparisons) as well as the NDLVC group (*p* = 0.009 and *p* < 0.00012, respectively) and in the NDLVC group compared to the NOCM group (*p* = 0.009 and *p* = 0.003, respectively).

LGE was acquired in all patients, except for nine (8%) NDLVC patients (with LV dysfunction without LV dilation) because of severe renal failure. As per definition, all patients in the NOCM group were LGE-negative, and, compared to this group, both NDLVC and DCM groups showed a significantly higher frequency of replacement myocardial fibrosis (*p* < 0.0001 for both comparisons). No patients showed an ischemic pattern. No difference between the NDLVC and DCM groups was found in the frequency of replacement myocardial fibrosis (*p* = 0.081), septal involvement (87.9% vs. 100%, *p* = 0459), and extent of LGE areas (1.89 ± 1.43% vs. 1.42 ± 0.36%, *p* = 0.838).

Patients without and with LGE showed comparable global heart T2* values (28.06 ± 12.16 ms vs. 27.29 ± 12.54 ms, *p* = 0.645), LV EDVI (84.07 ± 17.04 ms vs. 89.06 ± 19.59 ms, *p* = 0.060), and LV EF (62.70 ± 5.75% vs. 62.33 ± 7.11%, *p* = 0.725).

### 3.5. Outcome Analysis

The mean follow-up time was 57. 03 ± 18.01 months (median = 54.52 months).

Cardiac events were recorded in 32 (7.7%) patients: 15 episodes of HF (two with preserved ejection fraction), 15 arrhythmias (all supraventricular, including atrial fibrillation and atrial flutter), and 2 PH.

The mean time from the CMR scan to the development of a cardiac event was 22.07 ± 19.08 months (range 1–67 months). The mean age at the appearance of the cardiac events was 35.73 ± 7.37 years.

**Figure 2:**
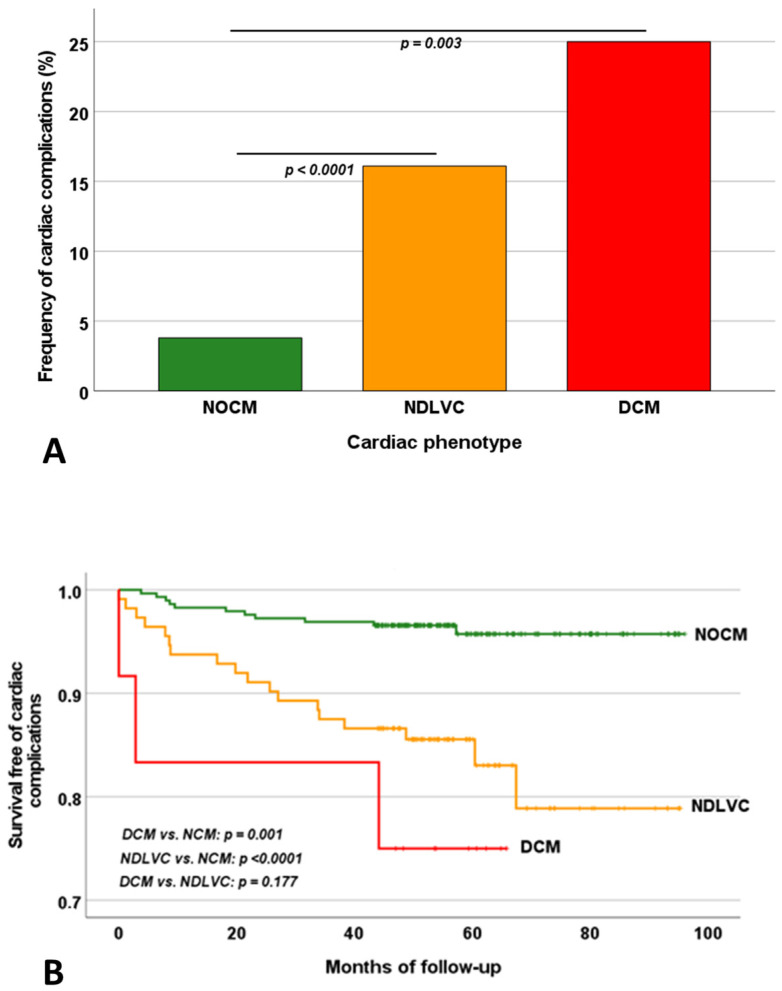
(**A**). Frequency of cardiac events in patients stratified according to the cardiac phenotype. The horizontal lines indicate a significant difference between the two groups. (**B**). Kaplan–Meier curves for cardiac events-free survival in patients stratified according to the cardiac phenotype. NOCM = no overt cardiomyopathy, NDLVC = non-dilated left ventricular cardiomyopathy, DCM = dilated cardiomyopathy.

The occurrence of cardiac events was significantly higher in the DCM group than in the NOCM group (25.0% vs. 3.7%, *p* = 0.003) and in the NDLVC group than in the NOCM group (16.5% vs. 3.7%, *p* < 0.0001), while it was not significantly different between the DCM and NDLVC groups (Figure 2A).

Table 2 shows the results of the univariate Cox regression analysis for the prediction of cardiac complications. Among the more traditional parameters, age, male sex, splenectomy, diabetes, and significant MIO were identified as univariate prognosticators. Compared to the NOCM group, both the NDLVC and the DCM groups were associated with a significantly increased risk of cardiac complications. The DCM did not provide additional risk in comparison to NDLVC (HR = 2.34, 95%CI = 0.67–8.22, *p* = 0.186). The Kaplan–Meier curve showing the impact of the cardiac phenotype on the development of cardiac events is shown in Figure 2B. The log-rank test revealed a significant difference in the curves (*p* < 0.0001).

In the multivariate analysis, the independent predictive factors were age (HR = 1.12, 95%CI = 1.07–1.18, *p* < 0.0001), male sex (HR = 2.75, 95%CI = 1.30–5.81, *p* = 0.008), significant MIO (HR = 2.46, 95%CI = 1.14–5.32, *p* = 0.022), and presence of DCM and NDLVC versus the NOCM phenotype (HR = 3.52, 95%CI = 1.63–7.59, *p* = 0.001 and HR = 5.44, 95%CI = 1.44–20.61, *p* = 0.013; respectively).

### 3.6. Stratification Based on MIO

Patients were categorized into two groups based on the presence of MIO.

For patients without MIO (*n* = 285), the incidence of cardiac events was 2.3% in the NOCM group, 12.3% in the NDLVC group, and 50.0% in the DCM group (*p* < 0.0001). Compared to the NOCM group, both the NDLVC and the DCM groups were associated with a significantly increased risk of cardiac complications (HR = 5.14, 95%CI = 1.68–15.74, *p* = 0.004 and HR = 23.49, 95%CI = 4.46–123.92, *p* < 0.0001; respectively).

For patients without MIO (*n* = 130), the incidence of cardiac events was 7.7% in the NOCM group, 22.7% in the NDLVC group, and 12.5% in the DCM group (*p* = 0.061). Compared to the NOCM group, only the NDLVC group was associated with a significantly increased risk of cardiac complications (HR = 2.82, 95%CI = 1.02–7.82, *p* = 0.046).

### 3.7. Chelation Treatment

At the baseline CMR, all patients were chelated. No difference was found in the distribution of the chelation regimens among the NOCM, NDLVC, and DCM groups (Table 1).

70.8% of the patients changed the chelation regimen during the follow-up, that is, they underwent dose modification (18.4%) or switched to a different type of chelator (52.4%).

Patients who changed the chelation regimen had a significantly higher frequency of significant MIO than patients who maintained the same chelation regimen (37.0% vs. 22.0%; *p* = 0.005).

## 4. Discussion

In this large cohort of TDT patients, we found that the prevalence of a DCM or a NDLVC phenotype occurred in 2.8% and 25.6% of cases, respectively. Despite similar demographic and clinical characteristics, biventricular volumes and systolic function were significantly worse in DCM patients compared to both NDLVC and NOCM patients, but also in NDLVC patients compared to NOCM patients. Moreover, NDLVC and DCM showed a higher burden of MIO and a higher prevalence of LGE than NOCM. From a prognostic perspective, NDLVC and DCM patients experienced more adverse cardiovascular events than NOCM during follow-up. According to Cox multivariate analysis, age, male sex, MIO, and the presence of either a DCM or NDLVC phenotype were all independent prognostic predictors.

At variance with the general population, in this cohort of TDT patients, the diagnosis of DCM and NDLVC phenotypes was based on previously derived disease-specific cut-offs, stratified by age and gender, in order to correct for the biventricular eccentric remodeling caused by chronic anemia [39]. Overall, the extremely low prevalence of the DCM phenotype suggests that further LV dilation on top of the expected LV dilation due to chronic anemia is a rare event. The prevalence of a NDLVC phenotype was around 25%, suggesting that pathophysiological changes mostly act through LV dysfunction and fibrosis.

Both DCM and NDLVC patients presented a worse MIO, in accordance with its known major pathophysiological role in promoting adverse cardiac remodeling in TDT patients. In the initial phases, MIO can result in a reduction in ventricular size due to heightened stiffness in both the vasculature and the ventricles [64]. However, the ventricular systolic function often remains well preserved, leaving patients largely asymptomatic. As the condition progresses, MIO may cause ventricular enlargement and a deterioration in systolic function [25,65,66]. Finally, MIO can promote extracellular matrix remodeling and collagen deposition [67,68,69], which can be assessed with LGE imaging [70,71,72]. However, in line with previous studies, we failed to detect a correlation between LGE and cardiac iron and function [73,74,75]. The most plausible explanations include the presence of normal or only mildly abnormal global heart T2* values and LV EF in most patients and the fact that replacement fibrosis appears to be irreversible, unlike cardiac iron accumulation and dysfunction. On the other hand, the lack of correlation between LGE and LV volumes suggests that LV dilation, even when it reaches extreme values such as those typical of this population affected by chronic volume overload, does not seem to play a major role in interstitial remodeling.

After the publication of the 2023 ESC guidelines on cardiomyopathies [47], few studies investigated the clinical and prognostic characteristics of NDLVC patients compared to DCM patients. In a multicenter study on 462 patients with DCM (227) or NDLVC (235), the NDLVC phenotype was associated with a lower risk of sudden cardiac death or major ventricular arrhythmias than the DCM phenotype, and in either case, the presence of LGE further increased the arrhythmic risk [76]. On the other hand, another study on 363 patients with non-ischemic LV dysfunction (EF < 50%) showed a similar risk of sudden cardiac death or HF hospitalization between NDLVC and DCM patients, suggesting that both phenotypes require equivalent attention during follow-up [77]. In our study, both the NDLVC and the DCM phenotypes presented a significantly worse prognosis than NOCM patients, without any significant difference between the NDLVC and the DCM phenotypes. Several important differences should be highlighted between our TDT population and other cardiomyopathy cohorts: the presence of chronic anemia already exerts a major effect on LV dilation, making the presence of further LV dilation a relatively rarer phenotype; the presence of a very specific etiology (iron overload and anemic volume overload) and the availability of a specific etiological treatment (iron chelating agents) may explain the different prognosis, mirrored by the occurrence of supraventricular arrhythmias, heart failure and pulmonary hypertension instead of ventricular arrhythmias. In our population, we observed a relatively low incidence of heart failure, which can be attributed to the patient-specific adjustments to the chelation regimen prompted by T2* imaging. Indeed, patients who modified their chelation therapy (whether by altering the drug, dosage, or frequency) during the follow-up period were more likely to have significant MIO at baseline. Symptomatic heart failure typically manifests in the advanced stages of cardiac siderosis and early intervention with intensive iron chelation therapy has been shown to offer the potential for clinical reversal [78,79,80].

At variance with the 2023 ESC guidelines on cardiomyopathies, other nosographic categorizations of heart muscle disorders have been proposed, for example, distinguishing hypertrophic/restrictive (H/RC), dilated/hypokinetic (D/HC) and scarring/arrhythmogenic (S/AC) cardiomyopathies [81]. Whether different nosographic approaches, as well as the inclusion of other genetic and molecular data, can provide a better diagnostic and prognostic management of cardiomyopathies is still a matter of investigation.

### Limitations

The findings of our study should be considered in the context of several limitations.

Being a retrospective observational study conducted using historical data, our research is susceptible to biases from unmeasured factors. The data in the E-MIOT registry were entered by physicians according to the content of the available medical records, and some information may be missing. Data on diastolic function and brain natriuretic peptide (BNP) levels were available for a limited subset of patients. However, in the initial years of the MIOT project, routine BNP measurements were not conducted in the thalassemia center laboratories.

We did not assess myocardial deformation (strain), which may be a more sensitive marker of myocardial dysfunction than EF [82,83,84]. However, as per current guidelines, LVEF remains the standard measure for diagnosing DCM and NDLVC. Additionally, although feature-tracking (FT) CMR allows for the quantification of myocardial deformation on routine SSFP cine images, the necessary post-processing FT software was not available at all MIOT centers.

The patients did not undergo coronary angiography or coronary computed tomography to exclude CAD. However, TDT is characterized by a low prevalence of CAD [85,86,87], and none of our patients presented with an ischemic LGE pattern. LGE-CMR has emerged as a highly effective method for excluding obstructive CAD [88,89].

The small number of patients with DCM restricted the ability to draw definite conclusions.

## 5. Conclusions

In TDT patients, DCM and NDLVC were present in 2.8% and 25.6% of patients, respectively. NDLVC and DCM showed a higher burden of MIO, a higher prevalence of LGE, and a worse prognosis than NOCM. At multivariate analysis, age, male sex, MIO, and the presence of either a DCM or NDLVC phenotype were all independent prognostic predictors of cardiac complications. From a clinical perspective, CMR represents an invaluable tool to distinguish between physiological remodeling secondary to chronic anemia and pathological changes, provided that disease-specific reference values are used. Further, multicenter studies are needed to investigate the clinical and prognostic significance of these patterns of adverse remodeling in larger thalassemic populations over a longer period of time and the best therapeutic strategies to counteract or even reverse them.

## Figures and Tables

**Table 1:** Comparisons of demographic, clinical, and instrumental data between TDT patients without overt cardiomyopathy, with NDLVC, and with DCM.

	NOCM (*n* = 294)	NDLVC (*n* = 109)	DCM (*n* = 12)	*p*-Value
Age (years)	29.18 ± 8.99	30.05 ± 8.49	33.02 ± 9.01	0.236
Females, n (%)	164 (55.8)	54 (49.5)	5 (41.7)	0.373
Splenectomy, n (%)	154 (52.4)	67 (61.5)	12 (100.0)	0.002
Age at splenectomy (years)	11.64 ± 8.63	11.06 ± 8.93	12.60 ± 6.74	0.466
Age at start of regular transfusions (years)	1.69 ± 1.52	1.49 ± 1.18	1.50 ± 0.84	0.766
Age at start of chelation therapy (years)	5.36 ± 5.15	4.78 ± 4.37	4.89 ± 5.51	0.467
Chelation therapy, n (%)				0.517
Deferoxamine	100 (34.0)	49 (45.0)	5 (41.7)	
Deferiprone	61 (20.7)	19 (17.4)	3 (25.0)	
Deferasirox	62 (21.1)	16 (14.7)	1 (8.3)	
Combined DFO + DFP	38 (12.9)	16 (14.7)	1 (8.3)	
Sequential DFO/DFP	33 (11.2)	9 (8.3)	2 (16.7)	
Mean pre-transfusion hemoglobin (g/dL)	9.69 ± 0.65	9.78 ± 1.24	9.23 ± 0.67	0.139
Mean serum ferritin (ng/mL)	1639.18 ± 1462.14	1747.32 ± 1879.88	1428.25 ± 860.56	0.985
Past/active HCV infection, n (%)	191 (65.0)	75 (68.8)	9 (75)	0.623
Diabetes, n (%)	23/283 (8.1)	15/105 (14.3)	1 (8.3)	0.189
Overweight, n (%)	49 (16.7)	16 (14.7)	3 (25.0)	0.638
Global heart T2* values (ms)	29.16 ± 11.78	24.83 ± 12.95	19.12 ± 13.26	0.001
Significant MIO, n (%)	78 (26.5)	44 (40.4)	8 (66.7)	0.001
N. of segments with T2* < 20 ms	4.38 ± 5.84	6.45 ± 6.80	9.92 ± 7.57	0.001
LV EDVI (ml/m^2^)	82.25 ± 15.59	87.44 ± 16.09	129.78 ± 11.09	<0.0001
LV ESVI (ml/m^2^)	29.74 ± 7.55	36.08 ± 9.94	62.88 ± 14.76	<0.0001
LV SVI (ml/m^2^)	52.28 ± 9.66	51.65 ± 9.82	66.73 ± 8.93	<0.0001
LV mass index (g/m^2^)	57.30± 13.02	60.04 ± 12.16	75.68 ± 14.82	<0.0001
LV EF (%)	64.02 ± 4.53	59.30 ± 7.27	51.92 ± 7.54	<0.0001
RV EDVI (ml/m^2^)	79.32 ± 16.26	83.27 ± 17.32	121.95 ± 13.42	<0.0001
RV ESVI (ml/m^2^)	30.02 ± 8.99	34.09 ± 9.83	58.39 ± 15.48	<0.0001
RV SVI (ml/m^2^)	49.24 ± 10.48	48.78 ± 11.83	63.56 ± 11.41	<0.0001
RV EF (%)	62.34 ± 6.37	59.34 ± 6.99	52.83 ± 9.21	<0.0001
Left atrial area index (cm^2^/m^2^)	12.42 ± 2.27	13.45 ± 2.75	16.33 ± 3.37	<0.0001
Right atrial area index (cm^2^/m^2^)	11.89 ± 2.11	12.90 ± 2.41	16.12 ± 2.82	<0.0001
Replacement myocardial fibrosis, n (%)	0 (0.0)	66/100 (66.0)	4 (33.3)	<0.0001

NOCM = no overt cardiomyopathy, NDLVC = non-dilated left ventricular cardiomyopathy, DCM = dilated cardiomyopathy, *n* = number, DFO = deferoxamine, DFP = deferiprone, HCV = hepatitis C virus, MIO = myocardial iron overload, LV = left ventricular, EDVI = end-diastolic volume index, ESVI = end-systolic volume index, EF = ejection fraction, RV = right ventricular.

**Table 2:** Results of univariate Cox analysis for cardiac events.

	*n* (%) in Group	*n* (%) of Cardiac Events	Univariate Analysis	
			HR (95%CI)	*p*-Value
Age			1.08 (1.04–1.12)	<0.0001
Sex				
females	223 (53.7)	13 (5.8)	Reference	
males	192 (46.3)	19 (9.9)	2.12 (1.04–4.30)	0.038
Splenectomy				
no	182 (43.9)	9 (4.9)	Reference	
yes	233 (56.1)	23 (9.9)	2.21 (1.02–4.79)	0.044
Age at start of regular transfusions			0.86 (0.58–1.29)	0.474
Age at start of chelation therapy			1.06 (0.99–1.13)	0.108
Mean pre-transfusion hemoglobin			0.71 (0.39–1.28)	0.256
Mean serum ferritin			1.00 (1.00–1.00)	0.461
Past/active HCV infection				
no	140 (33.7)	7 (5.0)	Reference	
yes	275 (9.1)	25 (9.1)	1.99 (0.86–4.62)	0.106
Diabetes mellitus				
no	361/400 (90.2)	23 (6.4)	Reference	
yes	39/400 (9.8)	8 (20.5)	3.35 (1.49–7.53)	0.003
Significant MIO				
no	285 (68.7)	15 (5.3)	Reference	
yes	130 (31.3)	17 (13.1)	2.14 (1.07–4.29)	0.033
Cardiac phenotype				
NOCM	294 (70.8)	11 (3.7)	Reference	
NDLVC	109 (26.3)	18 (16.5)	4.26 (2.01–9.04)	<0.0001
DCM	12 (2.9)	3 (25.0)	8.81 (2.41–32.17)	0.001

*n* = number, HR = hazard ratio, CI = confidence intervals, HCV = hepatitis C virus, MIO = myocardial iron overload, NOCM = no overt cardiomyopathy, NDLVC = non-dilated left ventricular cardiomyopathy, DCM = dilated cardiomyopathy.

## Data Availability

The data underlying this article cannot be shared publicly due to privacy reasons. The data will be shared on reasonable request to the corresponding author.
